# Scheimpflug analysis of corneal power changes after hyperopic small incision lenticule extraction

**DOI:** 10.1186/s12886-021-02043-w

**Published:** 2021-07-20

**Authors:** Dan Fu, Jianmin Shang, Xiaoyu Zhang, Lingling Niu, Tian Han, Xingtao Zhou

**Affiliations:** 1 Eye Institute and Department of Ophthalmology and Vision Science, Eye & ENT Hospital, Fudan University, No.19 Baoqing Road, Xuhui District, Shanghai, 200031 China; 2 NHC Key Laboratory of Myopia (Fudan University); Key Laboratory of Myopia, Chinese Academy of Medical Science, Shanghai, 20031 China; 3 Shanghai Research Center of Ophthalmology and Optometry, Shanghai, 20031 China

**Keywords:** Cornea power, Hyperopia, SMILE, TCRP, CHM

## Abstract

**Purpose:**

To assess the ability of the Pentacam in predicting the corneal power after hyperopic small-incision lenticule extraction (SMILE).

**Methods:**

Twenty-five eyes of 22 patients underwent hyperopic SMILE were prospectively followed. All patients finished at least 6 months visit. Cornea power was obtained by Pentacam HR, in the format of mean keratometry (Km), equivalent keratometry (EKR) and total cornea refractive power (TCRP). Calculation of TCRP were centered on either the corneal apex or the pupil center within a ring or zone, giving a total of four different subtypes naming AR、AZ、PR、PZ. Clinical history method (CHM) was regarded as a gold standard and was compared with other cornea power parameters.

**Results:**

Center difference had no impact on the TCR*P* values (PR vs AR and PZ vs AZ, *P* > 0.05). Compared with CHM, no difference was found in Km, EKR 4.0 mm, EKR 4.5 mm, PR 3.0 mm, PR 4.0 mm, AR 3.0 mm and AR 4.0 mm. PR 4.0 mm showed the least difference with CHM (− 0.14 ± 1.03D, *P* > 0.05). The 95% limit of agreement (LOA) of the TCRPs and CHM was not close. The top two were PR 3.0 mm and PR 4.0 mm, LOA of which were − 2.20 to 1.84 D and − 2.18 to 1.68 D respectively. Central cornea thickness was correlated with error (TCRP – CHM) of PR 4.0 mm (*r* = 0.58, *P* = 0.003).

**Conclusions:**

The Pentacam topographer is an alternative method of measuring corneal power in eyes after hyperopic SMILE. The optimal options seem to be the TCRP (PR 4.0 mm). The agreement needs more verifications.

## Introduction

Cornea refractive surgery is getting wide acceptance by surgeons and patients around the world. Surgical methods are constantly updated, from mechanical knife to femtosecond laser [1–3]. A common problem among the cornea refractive surgery recipients is accurate prediction of the corneal power for future use in intraocular lens (IOL) calculation. Several methods have been proposed to solve this problem [4]. In case the preoperative information is missing, one reliable postoperative cornea power would be helpful in IOL calculation. Traditional methods such as keratometer and Placido disc assume the anterior to posterior curvature is a fixed value. Cornea refractive surgery alters the cornea shape, thus traditional methods may cause an error of keratometric refraction index [5, 6]. In contrast, Scheimpflug topography considers both anterior and posterior cornea layer and can be used to calculate overall refractive power [7, 8]. Promising results of the ray tracing have been reported in eyes after myopic photorefractive keratectomy (PRK), laser in situ keratomileusis (LASIK), and small incision lenticule extraction (SMILE) [9–13].

Different from myopia, hyperopia is corrected by increasing the central refractive power and reducing the peripheral refractive power. There are a few studies about cornea power change after hyperopic surgery, and most of them focused on hyperopic LASIK [11, 14–17]. Hyperopic LASIK used excimer laser to ablate mid-periphery cornea area. Compared with LASIK, hyperopic SMILE is a relative young cornea surgery, by creating a convex lens using femtosecond laser. Reinstein et al. have reported the promising refractive results of hyperopic SMILE [18]. In terms of cornea power prediction, it seems no published study exists.

This study aims to assess the ability of the Pentacam in predicting the corneal power in eyes after hyperopic SMILE.

## Methods

### Ethics

This prospective study was performed at Eye, Ear, Nose and Throat Hospital (EENT) of Fudan University, Shanghai, China. All enrolled patients were informed and signed a consent form. All procedures in the study get approval of ethic committee of EENT and adhered to the tenets of Helsinki.

### Subjects

Patients satisfying following conditions were enrolled:

Inclusion criteria: age ≥ 18 years old; + 0.5 D ≤ spherical equivalent (SE) ≤ + 6.0 D; stable refraction for more than 2 years; finish at least 6 months follow-up, corrected distance visual acuity (CDVA) ≥ 20/40.

Exclusion criteria: Patients with significant abnormal corneal morphology or confirmed keratoconus; history of intraocular surgery; other eye diseases except ametropia such as significant dry eye, cataract, corneal degeneration, et.al; mental disorders or systemic diseases.

### Examinations

Regular preoperative examinations were performed, including uncorrected distance visual acuity (UDVA), CDVA, subjective refraction, corneal topography, axial length, and fundus topography. When compared with cornea power, refraction was adjusted for the corneal plane by using a vertex distance of 12 mm [19].

Corneal topography was collected by professional technicians using Pentacam HR (oculus, Wetzlar, Germany). Corneal powers were recorded in following formats:

- **total cornea refractive power (TCRP)**: TCRP can be measured in four ways according to the center location (pupil center or cornea apex) and ways of measuring (ring or zone):
- PR: Taking the center of pupil as the center, the average value was calculated by ring.
- PZ: Taking the center of pupil as the center, the average value was calculated by zone.
- AR: Taking the corneal apex as the center, the average value was calculated by ring.
- AZ: Taking the corneal apex as the center, the average value was calculated by zone.

- **equivalent keratometry reading (EKR)**: EKR is displayed in the “Holladay EKR Detail Report” in Pentacam. The calculation formula is “EKR = 0.376/r_1_ – 0.03165/r_2_”, where r_1_ and r_2_ represent anterior and posterior corneal curvature respectively [20].
- **mean keratometry (Km)**: This is the mean value of maximum and minimum axial power within the central 3.0 mm area. Km is calculated by “Km = (n-n_0_)/r_1_”, where n is the traditional keratometric index of refraction (1.3375), n_0_ is the refraction index of air and r_1_ is the radius of the anterior cornea surface [12].
- **clinical history method (CHM):** CHM is regarded as the gold standard of predicting postoperative corneal power [21]. It is obtained by subtracting the refractive change from the preoperative keratometry (Km in the current study).

### Surgical procedure

All the operations were performed by the same surgeon (XTZ). The patients were asked to lie flat on the operating table after disinfection, and their eyes were fixed on the green dot of the central stroboscopic ring. The laser scanning time was about 35 s. The corneal cap diameter was set to be 8.8 mm with thickness being 120 μm. The optical zone was 6.2–6.3 mm, adding a transition area of 2 mm. The lenticule thickness is calculated by the software, and the thinnest thickness was 15 μm. After scanning, the lens was removed from a small incision with a diameter of 2.3 mm at 12 o’clock.

### Statistics

The statistical software was SPSS (version 22, IBM Corp, USA). Before data analysis, *Kolmogorov Smirnov* was used to test whether the data obeyed normal distribution. For continuous variables, *ANOVA* was used to analyze the differences between different groups. The least significant difference (LSD) test was used for multiple comparisons. Paired t test was used to compare preoperative and postoperative results. The *Bland-Altman* was used to test agreement between cornea powers and CHM. *Pearson* correlation analysis was used to detect correlated factors. *P* < 0.05 was considered to be statistically significant.

## Results

### Refractive outcomes

The average follow up time was 8.4 ± 3.4 months (6–12 months). The average age of enrolled patients was 33.6 ± 10.6 years (18–55 years). No serious adverse events occurred during and after operation. The basic information of the enrolled patients is shown in Table 1. The average change of spherical equivalent (SE) was 3.15 ± 1.26D till the last visit.

**Table 1:** Preoperative and postoperative refractive information of enrolled patients

SMILE for Hyperopia	
No. of people (eyes)	22 (25)
Preoperative SE (D)	3.47 ± 1.46
Preoperative AL (mm)	22.18 ± 0.70
Preoperative UDVA (logMAR)	0.37 ± 0.27
Preoperative CDVA (logMAR)	0.09 ± 0.09
Preoperative central corneal thickness (μm)	558.8 ± 35.2
Preoperative IOP (mmHg)	15.8 ± 2.6
Lenticule thickness (μm)	107.0 ± 27.2
Postoperative SE (D)	0.32 ± 0.93
Postoperative UDVA (logMAR)	0.15 ± 0.09
Postoperative CDVA (logMAR)	0.10 ± 0.10
Preoperative ACD (mm)	2.78 ± 0.37
Postoperative ACD (mm)	2.72 ± 0.35
∆SE (D)	3.15 ± 1.26

*SE* spherical equivalent, *AL* axial length, *UDVA* uncorrected distance visual acuity, *CDVA* best corrected distance visual acuity, *IOP* intraocular pressure, *Km* mean corneal curvature, *ACD* anterior chamber depth, ∆SE = preoperative SE – postoperative SE

### Corneal power

The postoperative TCRP values were shown in Table 2. Four methods showed no difference within 5 mm area. Since diameter of 6 mm, TCRP calculated in zone was greater than values calculated in ring. From the center to the surrounding area, TCRP showed a trend of first increasing and then decreasing. The highest TCRP value mainly located at 3-5 mm area. Center difference had no impact on the TCRP values (PR vs AR and PZ vs AZ, *P* > 0.05).

**Table 2:** The mean TCRP within a diameter of 1.0 to 8.0 mm in eyes after hyperopic SMILE

	PR (D)	PZ (D)	AR (D)	AZ (D)	F	P
Diameter (mm)						
1	44.60 ± 1.89	44.46 ± 1.89	44.75 ± 1.92	44.67 ± 1.95	0.10	0.96
2	45.12 ± 1.83	44.75 ± 1.87	45.18 ± 1.91	44.93 ± 1.92	0.26	0.85
3	45.63 ± 1.90	45.11 ± 1.84	45.57 ± 1.95	45.19 ± 1.91	0.45	0.72
4	45.70 ± 1.94	45.38 ± 1.86	45.48 ± 1.97	45.36 ± 1.91	0.16	0.92
5	45.04 ± 1.87	45.37 ± 1.85	44.72 ± 1.90	45.26 ± 1.89	0.56	0.65
6	43.45 ± 1.84	44.98 ± 1.82	43.15 ± 1.88	44.84 ± 1.83	6.26	0.00
7	41.32 ± 2.16	44.25 ± 1.77	41.20 ± 2.19	44.10 ± 1.78	17.22	0.00
8	40.16 ± 2.39	43.41 ± 1.83	39.90 ± 2.40	43.3 ± 1.77	18.00	0.00

PR: Taking the center of pupil as the center, the average value was calculated by ring

PZ: Taking the center of pupil as the center, the average value was calculated by zone

AR: Taking the corneal apex as the center, the average value was calculated by ring

AZ: Taking the corneal apex as the center, the average value was calculated by zone

After translation to cornea plane, the mean CHM was 45.88 ± 1.83 D. Taking the CHM as the gold standard, no difference was found in Km, EKR 4.0 mm, EKR 4.5 mm, PR 3.0 mm, PR 4.0 mm, AR 3.0 mm and AR 4.0 mm. PR 4.0 mm showed the least difference with CHM (− 0.14 ± 1.03D, *P* = 0.39). Km was the most correlated parameter with CHM (*r* = 0.9, *P* < 0.01), followed by AZ 5.0 mm (*r* = 0.885, *P* < 0.01) and AZ 4.0 mm (*r* = 0.883, *P* < 0.01). (Table 3).

**Table 3:** Comparisons and correlation between CHM and other cornea power parameters

Cornea power	Mean ± SD (D)	Difference vs CHM (D)	*P* value	Correlation Coefficient	*P* value
CHM	45.88 ± 1.83	/			
Km	45.56 ± 1.73	−0.32 ± 0.80	0.06	0.900	0.00
EKR4.0 mm	45.41 ± 1.74	−0.34 ± 1.04	0.13	0.823	0.00
EKR4.5 mm	45.50 ± 1.71	−0.25 ± 1.01	0.24	0.831	0.00
PR2.0 mm	45.12 ± 1.83	−0.76 ± 0.97	0.00	0.858	0.00
PR3.0 mm	45.63 ± 1.90	−0.25 ± 0.97	0.22	0.866	0.00
PR4.0 mm	45.70 ± 1.94	−0.14 ± 1.03	0.39	0.858	0.00
PR5.0 mm	45.04 ± 1.87	−0.84 ± 1.00	0.00	0.852	0.00
PZ2.0 mm	44.75 ± 1.87	−1.13 ± 1.10	0.00	0.824	0.00
PZ3.0 mm	45.11 ± 1.84	−0.76 ± 0.98	0.00	0.857	0.00
PZ4.0 mm	45.38 ± 1.86	−0.50 ± 0.93	0.01	0.873	0.00
PZ5.0 mm	45.36 ± 1.85	−0.51 ± 0.90	0.01	0.881	0.00
AR2.0 mm	45.18 ± 1.91	−0.69 ± 0.96	0.00	0.870	0.00
AR3.0 mm	45.57 ± 1.95	−0.31 ± 0.94	0.12	0.879	0.00
AR4.0 mm	45.48 ± 1.97	−0.39 ± 0.96	0.06	0.874	0.00
AR5.0 mm	44.72 ± 1.90	−1.16 ± 1.00	0.00	0.856	0.00
AZ2.0 mm	44.93 ± 1.92	−0.95 ± 1.05	0.00	0.850	0.00
AZ3.0 mm	45.19 ± 1.91	−0.68 ± 0.94	0.00	0.876	0.00
AZ4.0 mm	45.36 ± 1.91	−0.52 ± 0.91	0.01	0.883	0.00
AZ5.0 mm	45.26 ± 1.89	−0.61 ± 0.89	0.00	0.885	0.00

PR: Taking the center of pupil as the center, the average value was calculated by ring

PZ: Taking the center of pupil as the center, the average value was calculated by zone

AR: Taking the corneal apex as the center, the average value was calculated by ring

AZ: Taking the corneal apex as the center, the average value was calculated by zone

*CHM* Clinical history method

**Fig. 1:**
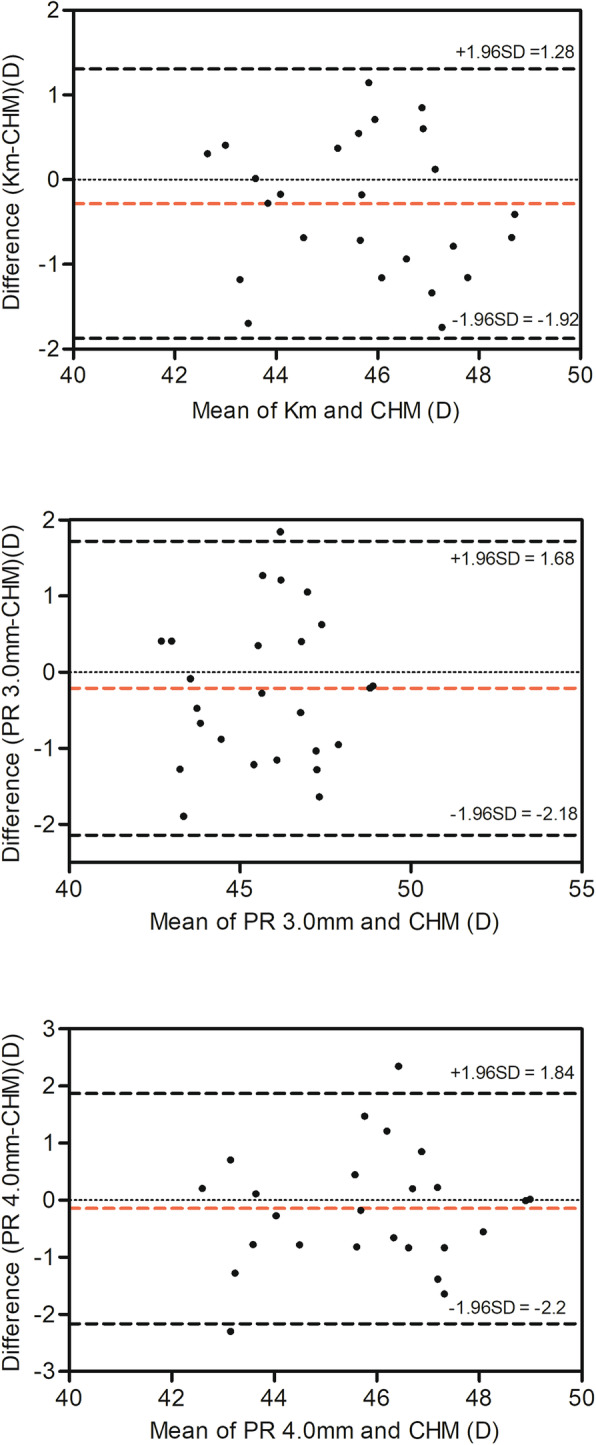
The mean vs difference plots comparing the cornea power parameters and CHM

Figure 1 showed the agreement between CHM and part of cornea power parameters. The 95% limits of agreement (LOA) of the Km and CHM was relatively high (− 1.92 to 1.28 D). The LOA of PR 3.0 mm and CHM, PR 4.0 mm and CHM were − 2.20 to 1.84 D and − 2.18 to 1.68 D respectively.

Since PR 4.0 mm showed the minimum error to CHM. Correlation analysis was performed between error PR 4.0 mm (PR 4.0 mm - CHM) and preoperative parameters (central cornea thickness [CCT], preoperative sphere, cylinder and intraocular pressure, maximum lenticule thickness). Results showed only CCT was correlated with error of PR 4.0 mm (*r* = 0.58, *P* = 0.003).

## Discussion

The current study demonstrated that the TCRP and EKR could potentially reflect the cornea power change after hyperopic SMILE.

The study found that all cornea power underestimated the CHM. The good predictor with minimum error was PR 4.0 mm (− 0.14 ± 1.03 D), followed by PR 3.0 mm (− 0.21 ± 0.99 D), EKR 4.5 mm (− 0.21 ± 1.02 D) and Km (− 0.28 ± 0.81D). It was reasonable that Km showed the highest correlation and narrowest LOA. Because in current study CHM was calculated based on Km. Km was one traditional keratometry calculating by thin-lens formula. It is equivalent to the simulated K of traditional corneal topographers, which was proved to be an accurate method to predict cornea power [22]. Our results demonstrated Km can be alternative of CHM after hyperopic SMILE.

Both EKR 4.0 mm and EKR 4.5 mm showed no difference with CHM, though the LOAs were relatively wide. The EKR was the same value measured by standard keratometry on the front surface, but considering the effect of the back surface power difference from normal. Holladay et al. found the optimal zone was EKR 4.5 mm in determining the IOL power. Compared with CHM, the average error was − 0.06 ± 0.56 D with range being − 1.63 to ±1.34 D [20]. In myopic LASIK, Alex et al. [10] demonstrated EKR 4.0 mm was the closest agreement with CHM. In myopic SMILE, Pan et al. [9] found EKR 4.0 mm and 4.5 mm showed no difference with CHM. The present study added the evidence of EKR after hyperopic SMILE.

In TCRP calculation, center location had no effect on TCRP values. Though hyperopia usually owes a higher kappa angle, our results were in agreement with previous studies [7, 9]. One reason was probably small sample size which lacks representation. Another interfering factor is pupil center may change with pupil size. Further studies in a larger population are warranted to explore this topic.

Among four methods, we found values at diameter 3.0 to 4.0 mm showed relative higher agreement. Previous studies have compared different curvature calculation methods, such as sim K, true net power and TCRP. All of them have come to the conclusion that TCRP is more suitable for actual refractive changes than only analyzing the axial refractive power of corneal surface [12, 14]. In hyperopic LASIK [14, 15], the TCRP 4.0 mm/5.0 mm zone calculation was proved to best predicted the surgically induced change in manifest refraction. Though their comparison standard is sphere equivalent change, not CHM in present study. Besides, the ablation method of LASIK is different from SMILE. The LASIK uses excimer laser ablates the peripheral cornea while retaining the central cornea (3 mm generally) unchanged, while the SMILE uses femtosecond laser to create complete stromal lens inside the corneal stroma, so the changes of corneal curvature should be different. This study takes the lead in analyzing the curvature changes of hyperopic SMILE, which may be of guiding significance for its clinical application and exploration.

Correlation analysis showed that preoperative corneal thickness affected the accuracy of TCRP in evaluating cornea power. The thinner the corneal thickness, the more likely TCRP underestimated the cornea power. The relationship between corneal thickness and corneal curvature is controversial. Some scholars believe that there is no correlation between corneal thickness and refraction and corneal curvature [23], while others hold the opposite opinion [24]. In addition, the accuracy of calculation may also be related to corrected refraction level, cornea aberration and other factors [25, 26]. Therefore, the inclusion of larger samples and more reference factors in the future is of great value to the accuracy of prediction.

One of the limitations is that the sample size is small and the influencing factors are not comprehensive enough, so the application of the results is limited, including the correlation with CHM may altered several years later. It should not be ignored that hyperopia SMILE has a relatively long recovery period, so longer-term postoperative corneal topography and curvature changes are still very valuable, and more factors should be considered, such as epithelial thickness, corneal aberration, etc. The above points can be continuations of present study in the future.

## Conclusions

The Pentacam topographer is an alternative method of measuring corneal power in eyes after hyperopic SMILE. The optimal options seem to be the TCRP (PR 4.0 mm). The agreement needs more verification.

## Data Availability

The datasets used and analyzed during the current study are available from the corresponding author on reasonable request.

## References

[1] Reinstein DZ, Archer TJ, Gobbe M (2012). The history of LASIK. J Refract Surg.

[2] Blum M, Lauer AS, Kunert KS, Sekundo W (2019). 10-year results of small incision Lenticule extraction. J Refract Surg.

[3] Kim TI, Alió Del Barrio JL, Wilkins M, Cochener B, Ang M (2019). Refractive surgery. Lancet.

[4] Rabsilber TM, Auffarth GU (2010). IOL power calculation after refractive surgery. Klin Monbl Augenheilkd.

[5] Mello GR, Roberts CJ, Smadja D, Serpe CC, Krueger RR, Santhiago MR (2013). Comparison of keratometric changes after myopic ablation: ray tracing versus simulated keratometry. J Refract Surg.

[6] Olsen T (1986). On the calculation of power from curvature of the cornea. Br J Ophthalmol.

[7] Naeser K, Savini G, Bregnhoj JF (2016). Corneal powers measured with a rotating Scheimpflug camera. Br J Ophthalmol.

[8] Koprowski R, Lanza M, Irregolare C (2016). Corneal power evaluation after myopic corneal refractive surgery using artificial neural networks. Biomed Eng Online.

[9] Pan C, Tan W, Hua Y, Lei X (2019). Comprehensive evaluation of total corneal refractive power by ray tracing in predicting corneal power in eyes after small incision lenticule extraction. PLoS One.

[10] Ng ALK, Chan TCY, Cheng ACK (2018). Comparison of different corneal power readings from Pentacam in post-laser in situ Keratomileusis eyes. Eye Contact Lens.

[11] Savini G, Calossi A, Camellin M, Carones F, Fantozzi M, Hoffer KJ (2014). Corneal ray tracing versus simulated keratometry for estimating corneal power changes after excimer laser surgery. J Cataract Refract Surg.

[12] Savini G, Hoffer KJ, Carbonelli M, Barboni P (2013). Scheimpflug analysis of corneal power changes after myopic excimer laser surgery. J Cataract Refract Surg.

[13] Savini G, Barboni P, Carbonelli M, Hoffer KJ (2013). Comparison of methods to measure corneal power for intraocular lens power calculation using a rotating Scheimpflug camera. J Cataract Refract Surg.

[14] Gyldenkerne A, Ivarsen A, Hjortdal JO (2015). Assessing the corneal power change after refractive surgery using Scheimpflug imaging. Ophthalmic Physiol Opt.

[15] Whang WJ, Yoo YS, Joo CK (2018). Corneal power changes with Scheimpflug rotating camera after hyperopic LASIK. Medicine.

[16] McNabb RP, Farsiu S, Stinnett SS, Izatt JA, Kuo AN (2015). Optical coherence tomography accurately measures corneal power change from laser refractive surgery. Ophthalmology.

[17] Wang L, Booth MA, Koch DD (2004). Comparison of intraocular lens power calculation methods in eyes that have undergone LASIK. Ophthalmology.

[18] Reinstein DZ, Pradhan KR, Carp GI, Archer TJ, Day AC, Sekundo W, Dhungana P (2019). Small incision Lenticule extraction for hyperopia: 3-month refractive and visual outcomes. J Refract Surg.

[19] Popov I, Valaskova J, Stefanickova J, Krasnik V (2017). Prevalence of refractive errors in the Slovak population calculated using the Gullstrand schematic eye model. Cesk Slov Oftalmol.

[20] Holladay JT, Hill WE, Steinmueller A (2009). Corneal power measurements using scheimpflug imaging in eyes with prior corneal refractive surgery. J Refract Surg.

[21] Savini G, Barboni P, Zanini M (2006). Intraocular lens power calculation after myopic refractive surgery: theoretical comparison of different methods. Ophthalmology.

[22] Awwad ST, Kelley PS, Bowman RW, Cavanagh HD, McCulley JP (2009). Corneal refractive power estimation and intraocular lens calculation after hyperopic LASIK. Ophthalmology.

[23] Sanchez-Tocino H, Bringas-Calvo R, Iglesias-Cortinas D (2007). Correlation between intraocular pressure, paquimetry and keratometry in a normal population. Arch Soc Esp Oftalmol.

[24] Ucakhan OO, Gesoglu P, Ozkan M, Kanpolat A (2008). Corneal elevation and thickness in relation to the refractive status measured with the Pentacam Scheimpflug system. J Cataract Refract Surg.

[25] Hamed AM, Wang L, Misra M, Koch DD (2002). A comparative analysis of five methods of determining corneal refractive power in eyes that have undergone myopic laser in situ keratomileusis. Ophthalmology.

[26] de Ortueta D, Arba-Mosquera S, Baatz H (2008). Topographic changes after hyperopic LASIK with the SCHWIND ESIRIS laser platform. J Refract Surg.

